# VALIDATION OF A WALKING SPEED MEASUREMENT DEVICE: GAITBOX

**DOI:** 10.1093/geroni/igz038.031

**Published:** 2019-11-08

**Authors:** Leighanne Jarvis, Matthew J Peterson, Miriam C Morey, Kevin Caves

**Affiliations:** 1 Duke University, Durham, North Carolina, United States; 2 Campbell University, Buies Creek, North Carolina, United States; 3 Duke Center for Aging/OAIC & Durham VA GRECC, Durham, North Carolina, United States

## Abstract

The NIH 4m Walk Test is a clinically validated tool to measure adult walking speed. Human reaction limitations can contribute to measurement error when manually timing gait speed. This is important considering a 0.10m/s decrease in walking speed is associated with a 12% decrease in life expectancy for older adults. The goal of this study was to validate a low cost, custom built device, Gait Box (GB), compared to human timer (HT) and a research grade Sprint Timing System (STS) with an older adult (mean 72.4 + 7.4 years of age) population (N = 35). Validity was assessed via accuracy (correlations), precision (mean differences), and bias (Bland-Altman plots). Results showed strong correlations between the GB and HT (0.99) and the GB and STS (0.98), with negligible mean differences. This demonstrates the GB can be used to accurately and precisely measure gait speed in clinical and research settings.

